# Extracellular vesicle microRNAs are biomarkers of focal epilepsy but not epilepsy‐related respiratory dysfunction

**DOI:** 10.1111/epi.18641

**Published:** 2025-09-18

**Authors:** Sylvain Rheims, Hayet Kouchi, Florence Busato, Stanislas Lagarde, David Derbala, Sébastien Boulogne, Mathilde Leclercq, Jessica Chenais, Sandrine Bouvard, Fabrice Bartolomei, Laurent Bezin, Jorg Tost

**Affiliations:** ^1^ Department of Functional Neurology and Epileptology Hospices Civils de Lyon and Lyon 1 University Lyon France; ^2^ Lyon Neuroscience Research Center INSERM U1028/CNRS UMR 5292/Lyon 1 University Lyon France; ^3^ Laboratory for Epigenetics & Environment, Centre National de Recherche en Génomique Humaine, CEA–Institut de Biologie François Jacob Université Paris‐Saclay Evry France; ^4^ Epileptology and Cerebral Rhythmology Assistance‐Publique Hôpitaux de Marseille, Timone Hospital Marseille France; ^5^ INSERM, Institute Neurosciences des Systèmes Aix Marseille Université Marseille France

**Keywords:** circulating miRNA, epilepsy, postictal hypoxemia, serotonin, SUDEP

## Abstract

**Objective:**

This study was undertaken to evaluate the diagnostic value of a set of preselected candidate microRNAs (miRNAs) extracted from plasma‐based extracellular vesicles (EVs) to identify patients with seizure‐related respiratory dysfunction.

**Methods:**

A two‐step design was applied. Step 1 entailed selection of the relevant miRNAs based on the combination of a literature review and an exploratory study in epileptic rats with or without interictal respiratory dysfunction. Step 2 involved evaluation of the diagnostic value of this preselected panel of circulating exosomal miRNAs in a case–control study conducted in 25 healthy subjects and 50 patients with drug‐resistant focal epilepsy undergoing video‐electroencephalographic (EEG) monitoring. Based on video‐EEG data, patients were separated into two groups: those with ictal/postictal hypoxemia (PIH; *n* = 24) and those without (noPIH; *n* = 26). Blood samples were collected in the interictal period (>24 h after the last seizure). Expression level of each miRNAs in EVs was compared (1) between all patients with epilepsy and controls and (2) between PIH and noPIH. Receiver operating characteristic (ROC) curves were generated, and the area under the curve (AUC) was calculated.

**Results:**

Following Step 1, the final set of miRNAs selected for evaluation in the case–control study included 24 miRNAs, with nine selected from published data in patients because of their potential regulatory role in the serotoninergic pathway, brain response to hypoxia, or epilepsy and 15 selected from the preclinical study in epileptic rats. Three miRNAs significantly differed between patients with epilepsy and controls (ROC curve AUC: hsa‐miR‐22‐3p, .74 [95% confidence interval (CI) = .63–.85]; hsa‐miR‐106b‐5p, .69 [95% CI = .57–.82]; and hsa‐miR‐26a‐5p, .72 [95% CI = .58–.85]). Only a trend toward higher expression levels was observed for hsa‐miR‐140‐3p in PIH compared to noPIH (+5%, *p* = .064).

**Significance:**

Whereas three miRNAs were robustly associated with epilepsy, none was significantly associated with seizure‐related respiratory dysfunction. Additional studies are required, including analysis of the expression of plasmatic cell‐free miRNAs, especially the miRNAs associated with interictal respiratory dysfunction in epileptic rats.

**Key points**

- Whether circulating miRNAs extracted from plasma‐based extracellular vesicles could help to identify patients at risk of complications during a seizure was investigated.
- Eleven miRNAs differed between epileptic and control rats and/or between epileptic rats with or without interictal respiratory dysfunction.
- Expression in extracellular vesicles of hsa‐miR‐22‐3p, hsa‐miR‐106b‐5p, and hsa‐miR‐26a‐5p was robustly associated with epilepsy.
- None of the 24 included miRNAs in the panel significantly differed between patients with or without ictal/postictal hypoxemia.
- Additional studies of the expression profile of plasmatic cell‐free miRNAs are required.

## 1 INTRODUCTION

Sudden unexpected death in epilepsy (SUDEP) represents the major cause of premature deaths in patients with drug‐resistant epilepsy.^1^ Although the exact pathophysiological mechanisms leading to SUDEP remain unknown,^2^, ^3^ experimental and clinical data strongly suggest that most SUDEP results from a postictal central respiratory dysfunction progressing to terminal apnea, followed by cardiac arrest.^1^ Although this hypothesis remains to be confirmed, it has been extensively suggested that identifying patients with seizure‐related respiratory dysfunction might help to better identify those at high risk of SUDEP.^4^ However, apart from directly recording central apnea and/or peri‐ictal hypoxemia (PIH) during a seizure, no marker has been validated to be associated with transient respiratory dysfunction.

MicroRNAs (miRNAs) are a class of endogenous small noncoding RNA molecules that are crucial for gene regulation.^5^ Several studies reported the link between miRNAs‐controlled pathways and epilepsy with a specific emphasis on the regulation of miRNAs‐mediated epileptogenesis.^6^, ^7^ Due to their high stability, cell‐free circulating miRNAs can be detected in the bloodstream and used as diagnostic tools.^8^ Although circulating miRNAs have been primarily evaluated as biomarkers for cancer, encouraging results in brain insults and/or brain diseases have also been reported. In the situation of brain injury, the pool of miRNAs might originate from controlled release in exosomes or from damage or disruption of the blood–brain barrier, allowing passage of small quantities of brain‐expressed miRNAs. In epilepsy, several studies have investigated the diagnostic value of circulating miRNAs, in plasma or in exosomes, to identify patients with epilepsy,^9^ to discriminate patients with drug‐resistant seizures from those with well‐controlled epilepsy^10^, ^11^ or patients with generalized convulsive seizures in mesial temporal lobe epilepsy.^12^ Although these results validated the concept that studying circulating miRNAs might be informative in patients with epilepsy, they remained focused on the epileptic process itself. None of these studies investigated whether the same approach might be used to diagnose and/or monitor seizure‐related complications or comorbidities.

To address this issue, we performed a proof‐of‐concept study with the primary objective to evaluate the diagnostic value of a set of candidate miRNAs extracted from plasma‐based extracellular vesicles including exosomes to identify patients with seizure‐related respiratory dysfunction. miRNAs were selected based on their implication in the regulation of molecular pathways involved in respiratory regulation.

## 2 MATERIALS AND METHODS

### 2.1 Project design

**FIGURE 1:**
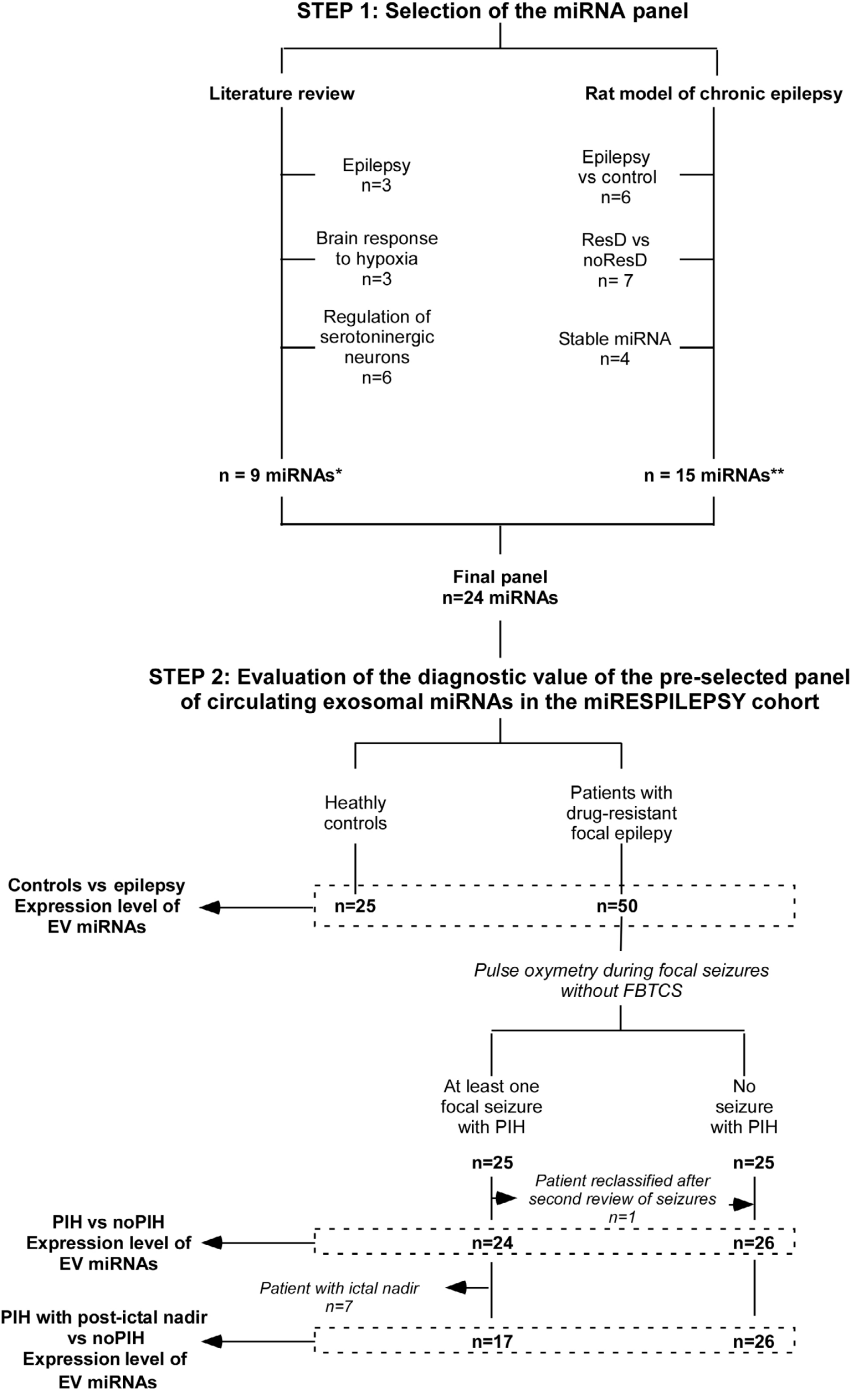
Flowchart of the study. *One microRNA (miRNAs; hsa‐miR‐22‐3p) is involved in the regulation of serotoninergic neurons and is also associated with brain response to hypoxia and epilepsy. **Expression level of two miRNAs (hsa‐miR‐223‐3p and hsa‐miR‐24‐3p) differed between epileptic rats and controls and between rats with long‐term interictal respiratory dysfunction (ResD) and those without respiratory dysfunction (noResD). EV, extracellular vesicle; FBTCS, focal to bilateral tonic–clonic seizures; PIH, peri‐ictal hypoxemia (SpO_2_ < 90%).

The primary objective of the miRESPILEPSY project was to evaluate the diagnostic value of a preselected panel of circulating exosomal miRNAs as biomarkers of epilepsy‐related respiratory dysfunction in patients with drug‐resistant focal epilepsy. To achieve this goal, we used a two‐step design (Figure 1):

1. Step 1: Selection of the relevant miRNAs based on the combination of a literature review and an exploratory preclinical study in a rat model of chronic epilepsy; and
2. Step 2: Evaluation of the diagnostic value of this preselected panel of circulating exosomal miRNAs in patients with drug‐resistant focal epilepsy.

Our methodological choice relied on the following elements: (1) in contrast to most previous studies, which were exploratory, we proposed a hypothesis‐driven selection of miRNAs based on the potential pathophysiology of epilepsy‐related respiratory dysfunction; (2) with genome‐wide profiling, the number of candidate miRNAs is typically high, requiring that the differences between expression levels are sufficiently pronounced to remain significant after correction for multiple comparisons or require the inclusion of a very large number of patients. Because the pool of brain‐expressed miRNAs is small, this issue might particularly be relevant in studies that aim to identify biomarkers of brain diseases, including epilepsy. Considering the complexity of collecting blood samples in combination with multimodal video‐electroencephalography (EEG), including identification of seizure‐related respiratory dysfunction, our proof‐of‐concept study could not be conducted in a large population of patients, resulting in an important risk of an underpowered analysis because of insufficient sample size.

### 2.2 Step 1: Selection of the miRNAs panel

As detailed in Text S1, Table 1, and Figure 1, the selection of relevant miRNAs was based on the combination of two selections: (1) a literature review and (2) an exploratory study in a rat model of chronic epilepsy.^13^, ^14^

**TABLE 1:** miRNAs panel.

miRNAs previously identified as potential circulating biomarkers in patients (cell free and/or extracellular vesicles)	Psychiatric diseases/response to serotoninergic drugs	Brain response to hypoxia	Epilepsy
hsa‐miR‐16‐5p	x		
hsa‐miR‐21‐5p		x	
hsa‐miR‐22‐3p	x	x	x
hsa‐miR‐106b‐5p			x
hsa‐miR‐135a‐5p	x		
hsa‐miR‐146a‐5p	x		
hsa‐miR‐146b‐5p	x		
hsa‐miR‐199a‐5p			x
hsa‐miR‐1202	x		

miRNAs identified in the present study in the rat model of chronic epilepsy	Epilepsy vs. controls	Epilepsy‐related respiratory dysfunction	Stable miRNAs expressed in rat EVs
hsa‐miR‐103a‐3p	x		
hsa‐let‐7f‐5p	x		
hsa‐let‐7 g‐5p	x		
hsa‐miR‐342‐3p	x		
hsa‐miR‐223‐3p	x	x	
hsa‐miR‐24‐3p	x	x	
hsa‐miR‐126‐3p		x	
hsa‐let‐7d‐5p		x	
hsa‐miR‐191‐5p		x	
hsa‐miR‐23a‐3p		x	
hsa‐miR‐3150b‐5p		x	
hsa‐miR‐148b‐3p			x
hsa‐miR‐30d‐5p			x
hsa‐miR‐140‐3p			x
hsa‐miR‐26a‐5p			x

Abbreviations: EV, extracellular vesicle; miRNAs, microRNA.

#### 2.2.1 miRNAs subpanel selected from published data in patients

In line with the involvement of serotonin in the regulation of breathing,^15^ it has been suggested from rodent models of SUDEP^16^, ^17^ and from postmortem data from patients with epilepsy who died from SUDEP^18^ that alteration of the brainstem serotoninergic pathway might play a key role in epilepsy‐related respiratory dysfunction. Accordingly, our literature review was focused on miRNAs involved in the regulation of serotoninergic neurons, completed by miRNAs associated with brain response to hypoxia and miRNAs proposed as circulating markers of epilepsy and of drug resistance.

#### 2.2.2 Identification of miRNAs of potential interest from a rat model of chronic epilepsy

This part of the study was conducted in Sprague Dawley rats using the same methods as previously reported^13^, ^14^ and was approved by the ethics committee of the University Claude Bernard Lyon 1 (approval 2018090611052453_v2). Briefly, status epilepticus (SE) was induced by pilocarpine in 16 rats aged 7 weeks, leading to the occurrence of spontaneous recurrent seizures 2–3 weeks later, as determined by a visual monitoring protocol. Nine to 10 weeks post‐SE, epileptic rats underwent plethysmography to assess ventilation. Ventilatory variables included respiratory frequency, estimated tidal volume, and minute ventilation. According to the respiratory abnormalities previously reported in the same model,^13^, ^14^ we separated epileptic rats (EpiRats) into two groups: those with respiratory dysfunction (ResDs) and those without (noResDs).

Following respiratory phenotyping (10–11 weeks post‐SE), blood samples were collected in the lateral tail vein in six ResDs and six noResDs, as well as in three control rats. All samples were centrifuged (4°C, 1500 × *g*, 10 min) within 15 min of collection. Plasma was frozen in liquid nitrogen and stored at −80°C before being transferred to the Laboratory for Epigenetics & Environment (National Center for Research on Human Genomics [CNRGH], Evry, France). The mean ± SD final volume of plasma samples was 483 ± 93.7 μL.

The methods used for (1) isolation of extracellular vesicles and extraction of total RNA, (2) RNA library preparation, and (3) small RNA sequencing and bioinformatic analysis of small RNA sequencing data are detailed in the supplementary methods. Briefly, extracellular vesicles were isolated by membrane affinity using the ExoRNeasy Midi Kit (Qiagen) followed by total RNA extraction on a Qiacube liquid handler (Qiagen) using RNeasy MinElute spin columns (Qiagen). Small RNAs extracted from extracellular vesicles (EVs) were converted into barcoded cDNA libraries using the QIAseq miRNAs Library Kit (Qiagen). A modified version of the pipeline provided by Qiagen for use with the QiaSeq protocol was used to process raw data (fastq files to counts). Counts normalization to transcripts per million (TPM) was performed during postprocessing. A threshold of TPM > 10 was applied to filter out lowly expressed miRNAs and then assess whether there are any significant differences in the overall distribution of particular samples using principal component analyses and normalized count statistics. The filtered count file was used for the differential analysis using Deseq2 (v.1.6.3), and in‐house R scripts were used to interpret and evaluate the result.

### 2.3 Step 2: Evaluation of the diagnostic value of the preselected panel of circulating exosomal miRNAs in patients with drug‐resistant focal epilepsy

#### 2.3.1 Study design

The miRESPILEPSY study (NCT03419000) was a case–control study that aimed at evaluating the diagnostic value of the preselected circulating exosomal miRNAs in a cohort of patients with drug‐resistant focal epilepsy undergoing long‐term video‐EEG monitoring, in which patients who demonstrate ictal/postictal hypoxemia (cases) were compared with those without seizure‐related respiratory dysfunction (controls). To ensure interpretability of miRNAs data, and specifically to evaluate the impact of epilepsy per se on the expression profile of the selected miRNAs, the study also included a group of healthy subjects.

The study was approved by the ethics committee (CPP Sud‐Est III; 2017‐A03307‐46), and all participants gave written informed consent.

#### 2.3.2 Participant selection

##### Patients

Adult patients (≥18 years old) with drug‐resistant focal epilepsy according to the International League Against Epilepsy classification^19^ undergoing long‐term video‐EEG/stereo‐EEG (SEEG) monitoring were recruited in two French epilepsy monitoring units (Lyon, Marseille). Because several of the selected miRNAs were previously identified in patients with psychiatric disorders, patients with ongoing major depressive episode (as defined by a score ≥ 15 on the French version of the Neurological Disorders Depression Inventory for Epilepsy scale) and/or current panic disorder (as defined by a score ≥ 7 on the French version of the Generalized Anxiety Disorder seven‐item scale), confirmed by Mini‐International Neuropsychiatric Interview and/or ongoing treatment with selective serotonin reuptake inhibitors, were excluded.

##### Healthy subjects

Healthy subjects were adults without medical history, including absence of epilepsy and of other medical conditions, such as cardiovascular or respiratory diseases. As for patients with epilepsy, subject with symptoms of anxiety and/or depression as defined by a score ≥ 11 on the French version of the Hospital Anxiety and Depression Scale^20^, ^21^ and those with ongoing treatment with selective serotonin reuptake inhibitors were not eligible.

#### 2.3.3 Study conduct

All seizures included in the present study had been locally reviewed by an epilepsy expert (S.L. in Marseille and S.R. in Lyon) to assess the characteristic of the seizure (focal or focal to bilateral tonic–clonic seizure [FBTCS]) and the evolution of the SpO_2_ during the 3 min preceding seizure onset, the seizure itself, and the 5 min following the end of the seizure. As previously reported,^22^ the SpO_2_ signal synchronized with the video was carefully analyzed to determine the period(s) during which SpO_2_ was not informative because of the patient's movements or because the oximetry sensor was not appropriately positioned on the patient's finger.

Patients were included based on the characteristics of the seizures recorded during video‐EEG/SEEG, and blood samples were collected according to the following organization:

- Collection of blood samples on the last day of the video‐EEG monitoring and at least 24 h after the last seizure.
- Two groups of patients according to presence/absence of PIH:
  ○ PIH group: Patients whose SpO_2_ was <90% during at least 5 s within the course of the seizure and/or within the 5 min postictally in at least one recorded purely focal seizure, excluding FBTCS. Considering that 86% of FBTCS are associated with post‐ictal hypoxemia,^22^ including patients with SpO_2_ < 90% during an FBTCS in the present study would have resulted in unbalanced groups, with an overrepresentation of this seizure type in the group of patients with ictal hypoxemia/PIH. This imbalance would have limited the interpretability of the data. Accordingly, the occurrence of FBTCS with postictal hypoxemia was not an exclusion criterion, but the patient was included in the study only if another focal seizure without FBTCS was associated with PIH.
  ○ noPIH group: Patients in whom none of the recorded seizures was associated with ictal/peri‐ictal SpO_2_ < 90%. Patients in whom SpO_2_ could not be assessed for technical reasons (i.e., disconnection or removal of the pulse oximeter) in ≥20% of recorded seizures were not eligible for inclusion. For the reasons described above, patients who had demonstrated FBTCSs with post‐ictal hypoxemia and focal seizures without PIH were not included in the study.
- Healthy subjects were included to ensure sex and age matching in comparison with patients with epilepsy. Blood samples were collected during an outpatient visit.

Seven blood samples (4 mL each in five ethylenediaminetetraacetic acid and two dry tubes) were collected from each individual. All samples were centrifuged (4°C, 1500 × *g*, 10 min) within 15 min of collection. Plasma was frozen in liquid nitrogen and then stored locally at −80°C before being sent in batches and stored centrally in a labeled biobank (Neurobiotec, Centre de Ressources Biologiques, Hospices Civils de Lyon, Lyon). The samples were then sent to the Laboratory for Epigenetics & Environment at CNRGH, where the epigenetic analyses were performed.

#### 2.3.4 Analysis of expression profile of EV miRNAs by quantitative polymerase chain reaction in human samples

All details are provided in the supplemental methods. In brief, a reverse transcription was first performed for each sample using the miRCURY LNA RT kit (Qiagen). miRNAs expression profile was determined by quantitative polymerase chain reaction (qPCR) using the miRCURY LNA SYBR Green PCR kit (Qiagen). GenEx software (version 6, Exiqon) was used for the normalization of data and differential expression analysis. All miRNAs with C_q_‐values ≥ 35 of were removed from the analysis. As miRNAs selected as reference miRNAs from the rat model did not turn out to be stable, we applied a global mean normalization, using the global mean of all Cq values < 35 for normalization, which has previously been shown to outperform other normalization strategies in terms of better reduction of technical variation and more accurate appreciation of biological changes.^23^, ^24^

#### 2.3.5 Determination of the sample size

The sample size of the study has been calculated according to the requirements for sensitivity and specificity analysis of a screening test.^25^ Considering that 50% of included patients demonstrate SpO_2_ < 90% in at least one recorded seizure, a minimum sample size of 40 patients (including 20 cases and 20 controls) was required to achieve a minimum power of 80% to detect an increase in the sensitivity of a diagnostic test from .50 to .80, with a target significance level of .05. This minimum sample size is also sufficient to detect a change in the value of specificity from 50.0% to 80.0%, which only requires a minimum sample of 40 patients. Because we anticipated potential technical difficulties in sample processing for a few patients that might limit evaluation of the expression profile of miRNAs, we included 50 patients, 25 patients with ictal/postictal hypoxemia and 25 without. In addition, 25 healthy subjects were included to allow evaluation of the expression profile of the selected miRNAs under nonpathological conditions.

#### 2.3.6 Statistics

Two analyses for the expression level of each miRNAs included in the panel were conducted in parallel: (1) comparison between all patients with epilepsy and healthy subjects and (2) comparison between the two groups of patients (PIH/noPIH).

In both analyses, miRNAs expression levels were analyzed using logistic regression and Mann–Whitney test. Adjustments for multiple comparisons were made using the Bonferroni method.

For each miRNAs with differential expression levels between the two groups, receiver operating characteristic (ROC) curves were generated to assess its sensitivity and specificity, and the area under the curve (AUC) was calculated.

## 3 RESULTS

### 3.1 Step 1: Selection of the miRNAs panel

#### 3.1.1 miRNAs subpanel selected from published data in patients

Based on the literature review detailed in Text S1, we selected nine miRNAs previously identified as potential circulating biomarkers in patients with epilepsy or other diseases. Two of them were proposed as circulating markers of epilepsy and of drug‐resistance (hsa‐miR‐106b‐5p, hsa‐miR‐199a‐5p), and one has been associated with brain response to hypoxia (hsa‐miR‐21‐5p). The six other are involved in the regulation of serotoninergic neurons (hsa‐miR‐135a‐5p, hsa‐miR‐16‐5p, hsa‐miR‐1202, hsa‐miR‐1202, hsa‐miR‐146a/b‐5p, hsa‐miR‐22‐3p), with hsa‐miR‐22‐3p also associated with brain response to hypoxia and epilepsy (Table 1).

#### 3.1.2 Identification of miRNAs of potential interest from a rat model of chronic epilepsy

We investigated the miRNAs expression profile in the EVs obtained from the plasma of controls rats (*n* = 3) and EpiRats (*n* = 12), including ResDs (*n* = 6) and noResDs (*n* = 6). A total of 313 miRNAs were consistently expressed in EVs at the chosen threshold level (TPM > 10). Principal component analysis of the miRNAs expression patterns showed no global difference in miRNAs expression patterns across groups (Figure S1) but identified one outlier sample that was removed from further analysis.

As shown in Table 2, we first analyzed the expression of seven of the nine miRNAs selected from published data in patients. When controls and EpiRats were compared, only hsa‐miR‐146b‐5p, whose expression in blood cells has previously been reported to be associated with response to treatment in patients with depression,^26^ significantly differed across groups, with lower expression in EpiRats than in controls (log2 fold change = −.48, *p* = .04). In contrast, none of the selected miRNAs differed between ResDs and noResDs.

**TABLE 2:** Characteristics of participants.

Characteristic	Healthy subjects, *n* = 25	Patients with focal epilepsy		
		All, *n* = 50	PIH, *n* = 24	noPIH, *n* = 26
Sex				
Female, *n* (%)	11 (44)	23 (46)	10 (42)	13 (50)
Age (years), mean ± SD	33.6 ± 9.9	37.1 ± 11.6	40.6 ± 10.6	33.8 ± 11.7
Self‐questionnaires, mean score ± SD				
HADS	5.5 ± 3.1			
GAD‐7^**^		5.7 ± 4.6	4.7 ± 4.1	6.3 ± 5.0
NDDI‐E scale^**^		9.5 ± 2.6	9.4 ± 2.7	9.8 ± 2.5
Epilepsy				
Age at onset, years, mean ± SD		16.8 ± 10.7	20.9 ± 11.5	19.6 ± 17.0
Epilepsy duration, years, mean ± SD		20.2 ± 14.4	20.4 ± 11.2	19.7 ± 16.9
Localization, *n* (%)				
Temporal		38 (76)	19 (79)	19 (73)
Seizure burden over the past 3 months				
Monthly seizure frequency, median (range)		3 (.2–150)	3 (.2–30)	3 (1–150)
≥ 1 focal to bilateral tonic–clonic seizure, *n* (%)		11 (22)	7 (30.4)	4 (15.4)
Delay between last seizure and blood collection, h, median (range)		38.8 (4.1–318.4)	42 (22–115.6)	32.5 (4.1–318.4)
Peri‐ictal hypoxemia				
Nadir, %, median (range)			80 (44–89)	
Duration, s, median (range)^***^			60 (9–360)	

Abbreviations: GAD‐7, Generalized Anxiety Disorder seven‐item scale; HADS, Hospital Anxiety and Depression Scale; NDDI‐E, Neurological Disorders Depression Inventory for Epilepsy; PIH, peri‐ictal hypoxemia.

^**^ Data missing in four patients with PIH.

^***^ PIH duration could not be assessed in 3 patients.

We then further explored the relative expression of all other valid miRNAs (*n* = 313). After correction for multiple comparisons, the expression of seven miRNAs differed between controls and EpiRats (Table 3): hsa‐miR‐103a‐3p, hsa‐let‐7f‐5p, hsa‐let‐7g‐5p, hsa‐miR‐342‐3p, hsa‐miR‐223‐3p, hsa‐miR‐24‐3p and hsa‐let‐7d‐5p. Overall, their expression level was lower in EpiRats than in controls for all of them. When ResDs and noResDs were compared, we observed differences for seven miRNAs, including three of those that also differed between EpiRats and controls (hsa‐miR‐223‐3p, hsa‐miR‐24‐3p, hsa‐let‐7d‐5p). None of these associations remained significant after correction for multiple comparisons, probably due to the low number of individuals in the groups (Table 2). However, these seven miRNAs were still considered of potential interest.

#### 3.1.3 Final miRNAs panel

The final set of miRNAs that we selected for evaluation in the miRESPILEPSY cohort consisted of the following (Table 1):

- Nine miRNAs selected from published data in patients because of their potential regulatory role in the serotoninergic pathway, brain response to hypoxia, or epilepsy; and
- Fifteen miRNAs selected from the preclinical study, including the 11 miRNAs whose expression levels differed between controls and EpiRats and/or between ResDs and noResDs and four miRNAs whose expression was stable between these groups in the dataset.

### 3.2 Step 2: Evaluation of the diagnostic value of the preselected panel of circulating exosomal miRNAs in the miRESPILEPSY cohort

#### 3.2.1 Participant characteristics

The main characteristics of healthy subjects and patients with epilepsy are provided in Table 3. The sex ratio (44% and 46% women) as well as the mean age of participants (33.6 ± 9.9 and 37.1 ± 11.6 years) were similar in both populations.

**TABLE 3:** Extracellular vesicle miRNAs of potential interest in epileptic rats.

Selected miRNAs	Controls (*n* = 3) vs. EpiRats (*n* = 12)			ResD (*n* = 6) vs. noResD (*n* = 6)		
	Log2 fold change	*p*	Adjusted *p*	Log2 fold change	*p*	Adjusted *p*
miRNAs previously identified as potential circulating biomarkers in patients						
hsa‐miR‐16‐5p	.17	ns	ns	−.29	ns	ns
hsa‐miR‐21‐5p	.29	ns	ns	−.42	.09	ns
hsa‐miR‐22‐3p	−.14	ns	ns	.04	ns	ns
hsa‐miR‐106b‐5p	.02	ns	ns	0	ns	ns
hsa‐miR‐146a‐5p	.36	ns	ns	.12	ns	ns
hsa‐miR‐146b‐5p	−.48	.004	.04	.01	ns	ns
hsa‐miR‐135a‐5p	na	na	na	na	na	na
hsa‐miR‐199a‐5p	.67	ns	ns	.30	ns	ns
hsa‐miR‐1202	na	na	na	na	na	na
miRNAs selected from the rat model of chronic epilepsy						
hsa‐miR‐103a‐3p	−1.17	<.001	<.001	.14	ns	ns
hsa‐let‐7f‐5p	−.47	<.001	.003	.02	ns	ns
hsa‐let‐7g‐5p	−.72	<.001	<.001	.06	ns	ns
hsa‐miR‐342‐3p	−.91	<.001	<.001	.08	ns	ns
hsa‐miR‐223‐3p	−.81	<.002	.002	.45	.003	ns
hsa‐miR‐24‐3p	−.53	.003	.03	.41	<.001	ns
hsa‐miR‐126‐3p	.01	ns	ns	.33	.01	ns
hsa‐let‐7d‐5p	−.64	.003	.03	.37	.04	ns
hsa‐miR‐191‐5p	−.22	ns	ns	.54	.02	ns
hsa‐miR‐23a‐3p	.02	ns	ns	.33	.03	ns
hsa‐miR‐3150b‐5p	.05	ns	ns	−.81	.04	ns
hsa‐mir‐148b‐3p	−.05	ns	ns	−.20	ns	ns
hsa‐miR‐30d‐5p	.39	.009	.068	−.1	ns	ns
hsa‐miR‐140‐3p	.16	ns	ns	−.09	ns	ns
hsa‐miR‐26a‐5p	−.03	ns	ns	−.05	ns	ns

Abbreviations: EpiRat, epileptic rat; miRNAs, microRNA; noResD, EpiRats without respiratory dysfunction; ns, not significant; ResD, EpiRats with respiratory dysfunction.

Among the 50 patients with epilepsy, 26 were included in the noPIH group and 24 in the PIH group, as one patient considered to be in the PIH group at the time of blood collection had been reclassified as noPIH after the second review of recorded focal seizures. In average, 3.76 ± 3.53 focal seizures were collected per patient (range = 1–18). After exclusion of seizures without informative SpO_2_ for technical reasons (i.e., disconnection or removal of the pulse oximeter), the mean number of available seizures per patient was 3.26 ± 3.11 (range = 1–15), with no difference between PIH (2.92 ± 2.62, range = 1–13) and noPIH (3.58 ± 3.53, range = 1–15, *p* = .78). In the PIH group, PIH was observed in ≥50% of SpO_2_ seizures in all but one patient, with PIH observed in all informative seizures in 16 (67%). In the remaining patient, PIH was observed in only one of the 13 recorded seizures. The median duration of PIH was 60 s (range = 9–360), with a median nadir of 80% (range = 44–89). The hypoxemia was exclusively peri‐ictal in three patients and exclusively postictal in another. In all other patients (83%), hypoxemia started during the seizure and was maintained in the postictal period in the majority of seizures, with recovery of SpO_2_ ≥ 90% after a median delay of 36 s after the end of the seizure (range = 11–490). SpO_2_ nadir was observed during the course of all seizures in seven patients (29%).

Epilepsy characteristics did not differ between the two groups (Table 3). Although the number of patients who reported at least one FBTCS over the 3 months before video‐EEG was greater in the PIH group (30% vs. 15%), the difference was not statistically significant (*p* = .2).

#### 3.2.2 Blood collection

The timing of blood sampling significantly differed between patients and healthy controls (*p* < .001), with blood collection performed in the morning (9:00 a.m.–1:00 p.m.) in 33 patients (66%) but in only five controls (20%). After correction for multiple comparisons, the expression level of four miRNAs significantly varied according to the time of day when sampling occurred (hsa‐miR‐22‐3p, *p* = .005; hsa‐miR‐140‐3p, *p* = .032; hsa‐miR‐30d‐5p, *p* = .005; and hsa‐miR‐191‐5p, *p* = .010). In contrast, the timing was similar in PIH and noPIH groups (62.5% vs. 61% of morning blood collections).

The delay between the last seizure and blood collection was <24 h in six patients with epilepsy, including one (22 h) in PIH and five in noPIH (4.1, 5.1, 17.3, 23, and 23.4 h). However, the median delay did not differ between PIH and noPIH (median: 42 vs. 32.5, *p* > .2) and was not associated with changes in the miRNAs expression profile.

#### 3.2.3 Circulating EV miRNAs

##### Patients with epilepsy versus healthy subjects

**FIGURE 2:**
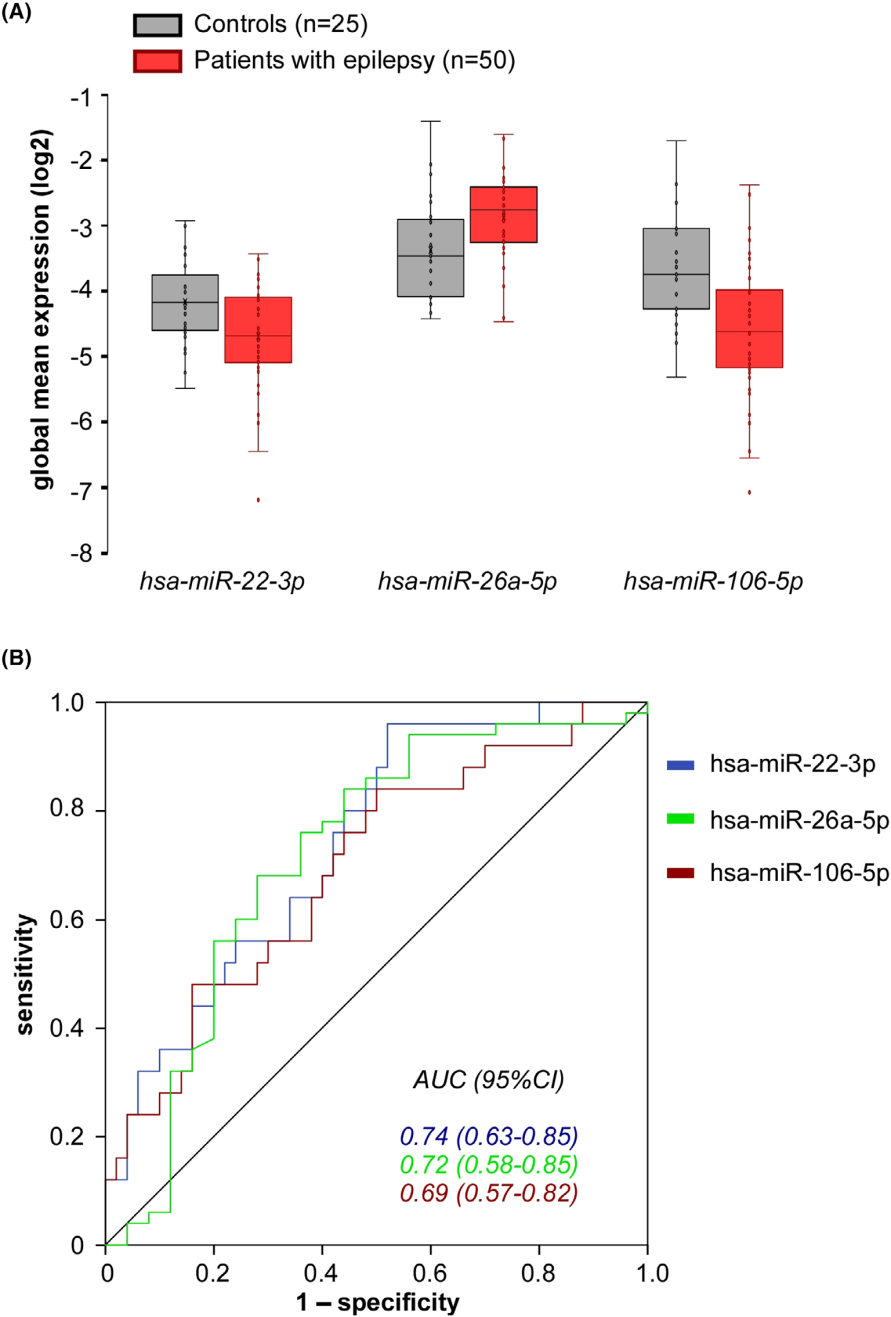
Expression in exosomes and other extracellular vesicles of hsa‐miR‐22‐3p, hsa‐miR‐106b‐5p, and hsa‐miR‐26a‐5p in healthy subjects and patients with epilepsy. AUC, area under the curve; CI, confidence interval.

Seven miRNAs differed between patients with epilepsy and controls. For three of them (hsa‐miR‐22‐3p, hsa‐miR‐106b‐5p, and hsa‐miR‐26a‐5p), the differences remained significant after correction for multiple comparisons (Table 4). Specifically, the expression level was lower in patients compared to controls for hsa‐miR‐22‐3p (−25%, *p* = .005) and hsa‐miR‐106b‐5p (−13%, *p* = .034), but greater for hsa‐miR‐26a‐5p (+15%, *p* = .034; Figure 2). The differences between patients with epilepsy and controls remained significant for the three miRNAs even after accounting for the timing of blood sampling. Notably, the expression level of hsa‐miR‐22‐3p was independently associated with both epilepsy (*p* = .018) and the timing of blood collection (*p* = .007, multivariate linear regression).

**TABLE 4:** miRNAs in extracellular vesicles in the miRESPILEPSY cohort.

Selected miRNAs	Patients with epilepsy vs. controls			Patients with vs. without PIH		
	Log2 fold change	*p*	Adjusted *p*	Log2 fold change	*p*	Adjusted *p*
miRNAs previously identified as potential circulating biomarkers in patients						
hsa‐miR‐16‐5p	1.09	ns	.328	1.03	ns	ns
hsa‐miR‐21‐5p	−1.11	ns	ns	1.02	ns	ns
hsa‐miR‐22‐3p	−1.25	.0002	.0053	1.09	ns	ns
hsa‐miR‐106b‐5p	−1.13	.0032	.0335	1.05	ns	ns
hsa‐miR‐146a‐5p	−1.03	ns	ns	1.02	ns	ns
hsa‐miR‐146b‐5p	1.03	ns	ns	−1.04	ns	ns
hsa‐miR‐135a‐5p	1.02	ns	ns	1.10	ns	ns
hsa‐miR‐199a‐5p	−1.04	ns	ns	1.04	ns	ns
hsa‐miR‐1202	−1.06	.0196	.1371	1.01	ns	ns
miRNAs selected from the rat model of chronic epilepsy					ns	ns
hsa‐miR‐103a‐3p	1.02	ns	ns	1.04	ns	ns
hsa‐let‐7f‐5p	−1.01	ns	ns	−1.02	ns	ns
hsa‐let‐7g‐5p	1.04	ns	ns	1.02	ns	ns
hsa‐miR‐342‐3p	−1.04	ns	ns	1.03	ns	ns
hsa‐miR‐223‐3p	1.82	ns	ns	2.37	ns	ns
hsa‐miR‐24‐3p	1.03	ns	ns	1.05	ns	ns
hsa‐miR‐126‐3p	1.17	.0511	.1596	−1.05	ns	ns
hsa‐let‐7d‐5p	1.06	.0311	.1371	−1.02	ns	ns
hsa‐miR‐191‐5p	−1.10	.0865	.2236	1.07	ns	ns
hsa‐miR‐23a‐3p	1.05	ns	ns	1.01	ns	ns
hsa‐miR‐3150b‐5p						
hsa‐miR‐148b‐3p	−1.05	.0354	.1371	−1.04	ns	ns
hsa‐miR‐30d‐5p	−1.08	.0515	.1596	1.05	ns	ns
hsa‐miR‐140‐3p	−1.07	.0252	.1371	1.05	.064	.*826*
hsa‐miR‐26a‐5p	1.15	.0024	.0335	−1.04	ns	ns

Abbreviations: miRNA, microRNA; PIH, peri‐ictal hypoxemia; ns, not significant.

ROC curves for these three miRNAs were very similar (Figure 2), with little difference in their AUCs: .74 (95% confidence interval [CI] = .63–.85) for hsa‐miR‐22‐3p, .69 (95% CI = .57–.82) for hsa‐miR‐106b‐5p, and .72 (95% CI = .58–.85) for hsa‐miR‐26a‐5p. To quantitatively assess the potential added diagnostic power of combining these three miRNAs in this study population, we performed a logistic regression analysis, with each of them as an independent variable and diagnosis of epilepsy as the dependent variable, and generated the ROC curve for their joint regression‐derived marker. The AUC of the combined signature, .75 (95% CI = .63–.87), did not differ from the one of each miRNAs, suggesting that the combination of these miRNAs did not provide additional information. No association was observed between the expression level of these miRNAs and participants' age, duration of epilepsy, localization of epilepsy (temporal vs. extratemporal), or seizure burden over the past 3 months (data not shown).

##### PIH versus noPIH

**FIGURE 3:**
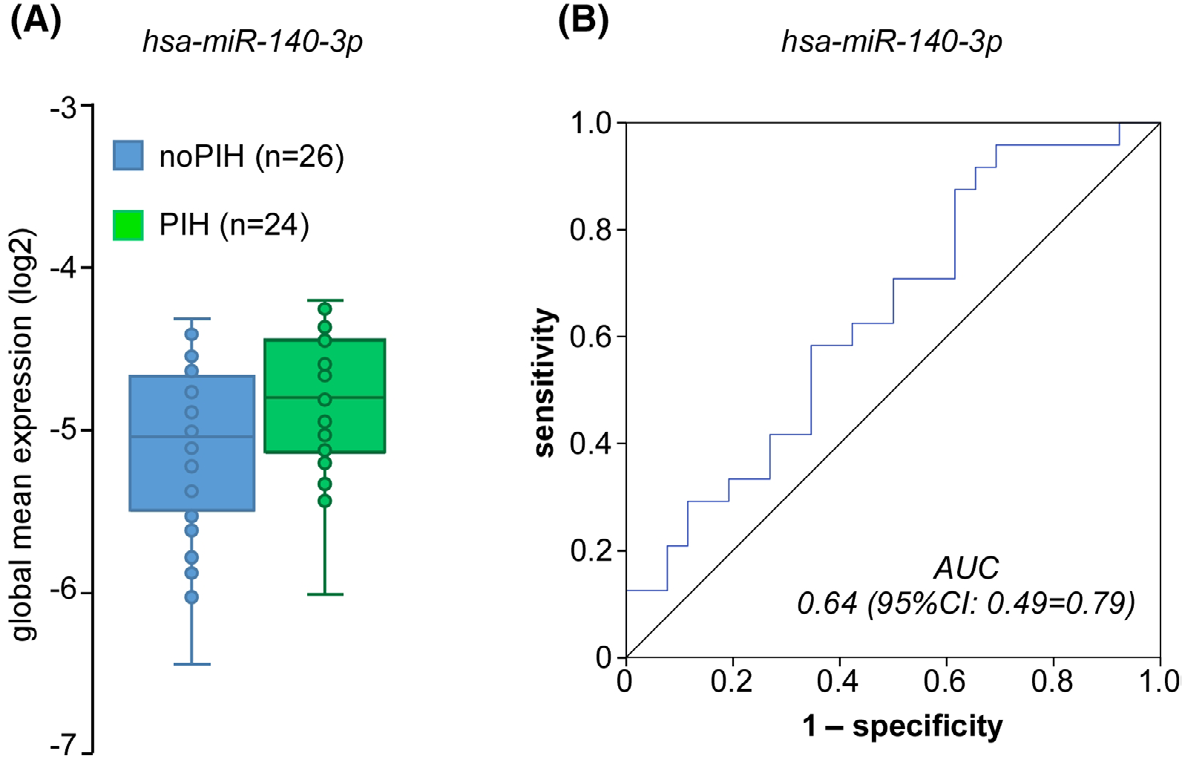
Expression level of hsa‐miR‐140‐3p in patients with (PIH) or without (noPIH) ictal/postictal hypoxemia. AUC, area under the curve; CI, confidence interval.

None of the 24 miRNAs included in the panel significantly differed between PIH and noPIH. We only observed a trend toward a higher expression level of hsa‐miR‐140‐3p in PIH compared to noPIH (+5%, *p* = .064 nominal *p*‐value without correction for multiple comparisons), with an ROC curve AUC of .64 (95% CI = .49–.79; Figure 3). The results remained similar when the timing of blood sampling was taken into account (*p* = .071). All analyses were reprocessed after exclusion from the PIH group of the seven patients whose SpO_2_ nadir was always observed in the ictal period. We also observed a trend toward a higher expression level of hsa‐miR‐140‐3p in PIH compared to noPIH (+6.6%, *p* = .057 nominal *p*‐value without correction for multiple comparisons), with an ROC curve AUC of .69 (95% CI = .52–.85), and with similar results when the timing of blood sampling was taken into account (*p* = .059).

## 4 DISCUSSION

Our study is the first to investigate whether the expression profile of miRNAs contained in exosomes and other extracellular vesicles measured in the interictal period could help to identify patients at risk of complications during a seizure, specifically those at risk of seizure‐induced respiratory dysfunction. Our study did not, however, identify exosomal miRNAs significantly associated with PIH in the miRESPILEPSY cohort, thus not achieving its primary objective. However, our study still provides informative data: (1) three exosomal miRNAs were robustly associated with epilepsy in comparison with healthy subjects; (2) although the association was weak, EV expression of hsa‐miR‐140‐3p might be lower in patients with PIH; and (3) despite the small sample size of the rodent part of the study, several miRNAs were associated with epilepsy and/or ResDs and might be worth further investigating.

The present study investigated a targeted set of 24 candidate miRNAs. Our method differed from the three‐step approach typically used to identify cell‐free miRNAs biomarkers, with the first identification of candidate miRNAs using genome‐wide profiling, followed by their validation by real‐time qPCR, before comparison of the expression levels of validated miRNAs in independent cohorts. Our methodological choice relied on the following elements: (1) a hypothesis‐driven selection of miRNAs based on the presumptive pathophysiology of epilepsy‐related respiratory dysfunction; and (2) considering the complexity of collecting blood samples in combination with multimodal video‐EEG, our proof‐of‐concept study could not be conducted in a large populations of patients, resulting in an important risk of an underpowered study because of insufficient sample size if we used genome‐wide profiling.

However, we cannot exclude that our approach was too narrow to identify relevant EV‐based miRNAs. Because we chose to select from published data only the miRNAs whose circulating profile had proven to be associated with a disease that involves a neurobiological pathway that might echo the presumptive pathophysiology of epilepsy‐related respiratory dysfunction, our panel was exposed to the risk of publication bias. Furthermore, because of the exploratory nature of the preclinical study, its sample size remained small, and this approach might have affected our ability to select the best miRNAs panel for the case–control study. In this context, a broader, unbiased screening approach directly in human samples might have yielded different insights. This issue might be reinforced when one considers that miRNAs are usually implicated in several regulatory processes. Most importantly, we mainly focused on miRNAs considered to be involved in the regulation of the serotonin pathway. However, the pathophysiology of epilepsy‐related respiratory dysfunction and SUDEP might involve other pathways, including endogenous opioids^27^ or the adenosine pathway.^28^, ^29^ Interestingly, hsa‐miR‐22‐3p, whose expression level differed in patients and healthy subjects, has been proposed as a surrogate of the P2X7 purinoceptor's brain expression in patients with mesial temporal lobe epilepsy.^30^ Furthermore, we did not investigate long noncoding RNAs, which are involved in the pathophysiology of epilepsy.^31^

Another limitation of our study was the difference of the time of day when sampling occurred between patients and controls, with collection predominantly in the morning in the former and in the afternoon in the latter. It has been reported that miRNAs expression can be influenced by circadian rhythms, including hsa‐miR‐22‐3p.^32^ The differences between patients with epilepsy and controls remained significant for the three miRNAs in the multivariate analysis. However, expression level of hsa‐miR‐22‐3p was also associated with time of blood sampling, suggesting that design of future studies should take into account the potential impact of circadian rhythms on results.

Previous studies showed that ictal hypoxemia in focal seizures is accompanied by central apnea, assessed either by direct measures of respiratory function with respiratory belts or by the simultaneous use of EtCO_2_ measurement as an index of hypoventilation.^33^, ^34^, ^35^ Although our approach focused on SpO_2_ measurements, integrating plethysmography or EtCO_2_ monitoring could have enhanced the assessment of seizure‐related respiratory dysfunction by providing additional insights into our investigations. Specifically, our assessment of seizure‐related respiratory dysfunction did not allow us to formally discriminate ictal and postictal apneas, although their pathophysiology, and association with risk of SUDEP, might be different.^36^ Considering the results after exclusion of patients whose SpO_2_ nadir was always observed in the ictal period, investigating the relation between hsa‐miR‐140‐3p and postictal apnea might be interesting. miR‐140‐3p might play a role in the regulation of oxygen–glucose deprivation/reoxygenation in stroke^37^ or in myocardial infarction.^38^ However, miR‐140‐3p might also be involved in microglial chemotaxis through the regulation of *ADAM10* and has been reported to be upregulated in the blood plasma of patients with Alzheimer disease.^39^

We only considered seizure‐related respiratory dysfunction during focal seizures. This methodological choice was driven by the data available at the initiation of the miRESPILEPSY project, without clear evidence at that time that the pathophysiology of peri‐ictal respiratory dysfunction during focal seizures was different from that triggered by FBTCSs.^4^ However, we could not exclude that investigating miRNAs expression with respect to occurrence and/or severity of FBTCS‐related respiratory dysfunction might have led to different results. This issue might be particularly important, because the frequency of generalized convulsive seizures is the main risk factor of SUDEP,^40^ with evidence that most SUDEP results from fatal postictal apnea.^41^ Considering the association between postictal apnea after generalized convulsive seizures and postictal serotonin levels,^42^ whether the expression level of miRNAs with a potential regulatory role in the serotoninergic pathway might differ between patients with or without postconvulsive centra apnea remains an open question.

Two of the three miRNAs that significantly differed in patients with epilepsy and healthy subjects, miR‐22‐3p and miR‐106b‐5p, had previously been identified as markers of epilepsy in previous studies.^9^, ^30^ Specifically, low miR‐22‐3p serum levels might be associated with poor response to antiseizure drugs in patients with temporal lobe epilepsy,^30^ and cell‐free miRNAs levels of miR‐106b were increased in patients with epilepsy in comparison with healthy controls.^9^ Surprisingly, in our cohort, the expression level of miR‐106b‐5p was lower in patients than in controls. In these previous studies, the expression profile of circulating miR‐22‐3p and miR‐106b‐5p was investigated in serum. By focusing on exosomal miRNAs, our study provides new data that reinforce the potential value of these two miRNAs in the circulation. Only a few studies have investigated miRNAs within extracellular vesicles and exosomes in patients with epilepsy.^43^, ^44^, ^45^ None of the 10 miRNAs identified in these studies was included in our panel. In addition, none of the four that were investigated in rodent models (miR‐27a‐3p, miR‐328‐3p, miR‐155‐5p, and miR‐184.) significantly differed between EpiRats and controls, although the expression level of miR‐184 was higher in EpiRats compared to controls when the analysis was not corrected for multiple comparisons (log2 fold change = 1.59, *p* = .02).

Similarly, only one of the studies in other diseases from which our panel of miRNAs was selected analyzed exosomal miRNAs.^46^ miRNAs contained in extracellular vesicles only represent a small subset of the cell‐free miRNAs released into biofluids. However, whereas circulating miRNAs are mainly released from dying cells, especially blood cells, miRNAs contained in vesicles are part of a complex cell‐to‐cell signaling system and are actively selected by the producing cells for specific purposes and target cells. In this context, we therefore cannot exclude that the contribution of the exosomal miRNAs to the previously observed expression differences was minor and did not allow us to replicate significant differences across groups. It would therefore be of interest in the future to complement our analyses with the expression profile of plasmatic cell‐free miRNAs. This will allow us either to reinforce the present findings, including the lack of significant differences between PIH and noPIH, or to identify miRNAs associated with peri‐ictal respiratory dysfunction, including those preselected from the literature review. This complementary analysis might also permit extension of the study of circulating miRNAs as a marker of epilepsy severity or of epilepsy‐related respiratory dysfunction in other epilepsy syndromes at very high risk of SUDEP.

## FUNDING INFORMATION

The miRESPILEPSY study was funded by the French Foundation of Epilepsy Research (2018 clinical research grant). H.K. was funded by the French Chapter of the International League Against Epilepsy (Ligue Française contre l'Epilepsie, Research Award 2019). S.R. was funded by INSERM (Contrat d'interface Hospitalier 2018) and is funded by HORIZON‐ERC, award number ERC‐2023‐COG‐101125118 (EPIAROUSAL).

## CONFLICT OF INTEREST STATEMENT

None of the authors has any conflict of interest related to this study to disclose. We confirm that we have read the Journal's position on issues involved in ethical publication and affirm that this report is consistent with those guidelines.

## Supporting information


Figure S1.

Data S1.

## Data Availability

The data that support the findings of this study are available from the corresponding author upon reasonable request.
