# Supplementary figures and images for: Extracellular vesicle microRNAs are biomarkers of focal epilepsy but not epilepsy‐related respiratory dysfunction
Source: Epilepsia. 2025 Sep 18;67(1):408–23. doi: 10.1111/epi.18641 (PMC12893299; doi:10.1111/epi.18641)

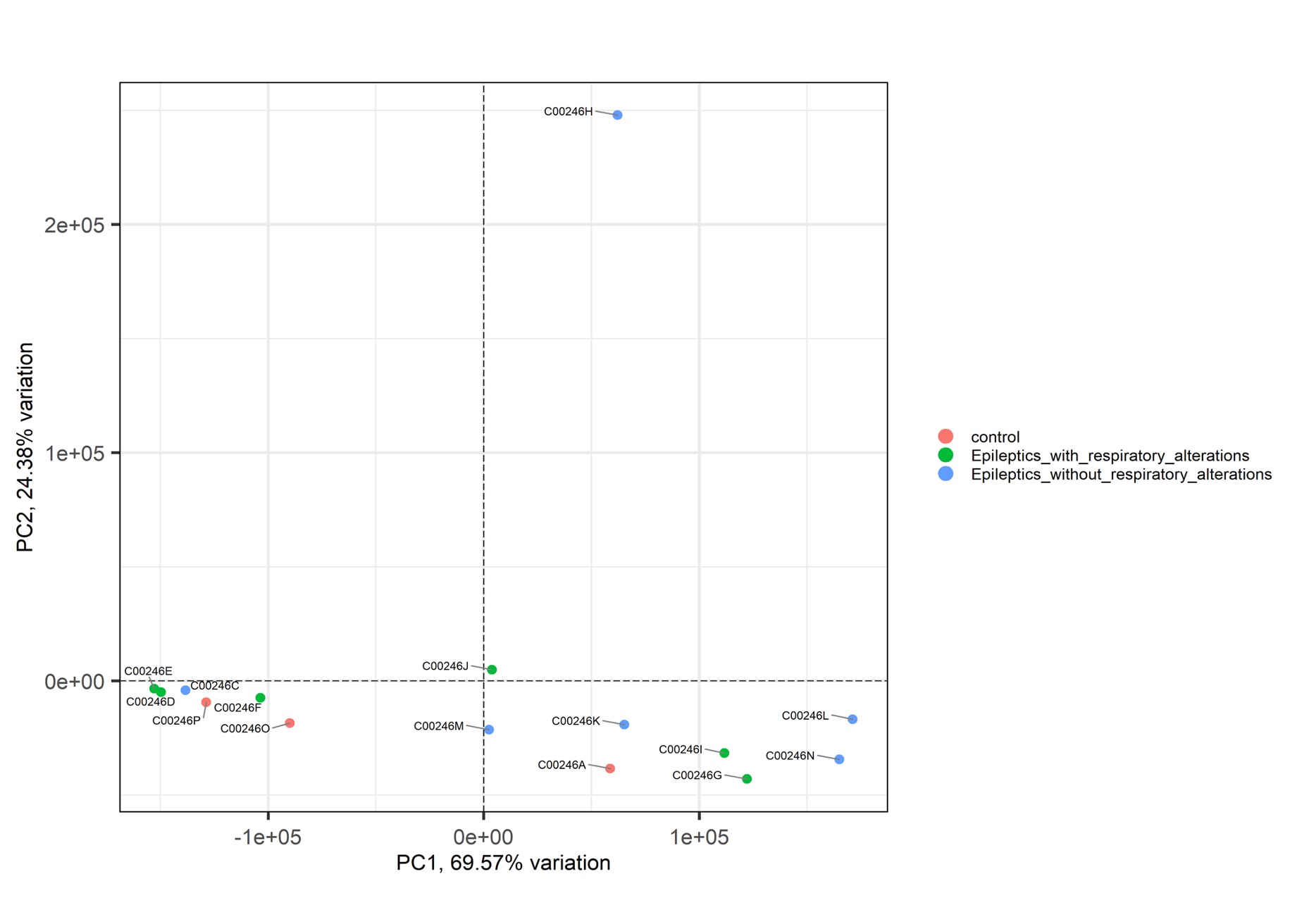

Supplement: Supplementary file 1 — Figure S1. [file EPI-67-408-s002.jpg]
