# Supplementary material for: Extracellular vesicle microRNAs are biomarkers of focal epilepsy but not epilepsy‐related respiratory dysfunction
Source: Epilepsia. 2025 Sep 18;67(1):408–23. doi: 10.1111/epi.18641 (PMC12893299; doi:10.1111/epi.18641)
Supplement: Supplementary file 2 — Data S1. [file EPI-67-408-s001.docx]

**Supplemental Text S1: Detailed methods**

1. **Selection of the miRNAs panel**

The present study investigated a targeted set of 24 miRNAs. The selection was based on the combination of three sub-panels, as detailed in Table 1 and Figure 1.

This method differed from the three-step approach typically used to identify circulating miRNAs in previous studies; with first identification of candidate miRNAs using genome-wide profiling, followed by their validation by real-time qPCR. Our methodological choice relied on the following elements: (i) in contrast to most of previous studies which were exploratory, we proposed a hypothesis-driven selection of miRNAs based on the potential pathophysiology of epilepsy-related respiratory dysfunction; (ii) with genome-wide profiling, the number of candidate miRNAs is typically high, requiring that the differences between expression level are sufficiently pronounced to remain significant after correction for multiple comparisons or require the inclusion of a very high number of patients. Because the pool of brain-expressed miRNA is small, this issue might particularly be relevant in studies which aim to identify biomarkers of brain diseases, including epilepsy. Considering the complexity of collecting blood samples in combination with multimodal video-EEG, including identification of seizure-related respiratory dysfunction, our proof-of-concept study could not be conducted in a large population of patients, resulting in an important risk for an underpowered analysis because of insufficient sample size.

The 24 miRNAs investigated in the present study were selected based on (i) a literature review to identify miRNAs which have previously been identified as potential circulating biomarkers in other brain diseases, ensuring that disease-related variations in their circulating expression can be detected in certain conditions, and whose known molecular targets are involved in cerebral molecular pathways related to central respiratory regulation ; (ii) an exploratory study in a rat model of chronic epilepsy, in which we previously showed that evolution of epilepsy is associated with long-term interictal respiratory dysfunction in about 40% of epileptic rats ^(1; 2)^. These two sub-panels were completed by set of miRNAs required to ensure the quality control of the analyses as well as for normalization.

- 1. miRNAs sub-panel selected from published data in patients because of their potential regulatory role (Table 1)

In line with the involvement of serotonin in the regulation of breathing ^(3)^, it has been suggested from rodent models of SUDEP ^(4; 5)^ and from post-mortem data from patients with epilepsy who died of SUDEP^(6)^, that alteration of the brainstem serotoninergic pathway might play a key role in epilepsy-related respiratory dysfunction. Furthermore, it has been suggested from blood analyses in patients undergoing long-term video-EEG that postictal serotonin levels might be associated with peri-ictal apnea ^(7)^. Accordingly, our selection was focused on miRNAs involved in the regulation of serotoninergic neurons, completed by miRNA associated with brain response to hypoxia and miRNAs proposed as circulating markers of epilepsy and of drug-resistance.

- - **hsa-miR-135a-5p:** expressed at relatively high levels in the midbrain raphe nuclei, regulates both the serotonin transporter (SERT) and serotonin receptor-1a transcripts (HTR1A) ^(8)^. In comparison with healthy subjects, blood levels of miR-135a were significantly reduced in patients suffering from moderate-to-severe depression ^(8)^
  - **hsa-miR-16-5p:** expressed in noradrenergic and serotoninergic neurons of the median raphe and acting as negative regulator of SERT translation ^(9)^. In patients with moderate-to-severe depression, there was a trend toward a reduction of blood levels of miR-16 in comparison with healthy subjects ^(8)^
  - **hsa-miR-1202:** regulates expression of the gene encoding the metabotropic glutamate receptor-4, modulating glutamatergic, dopaminergic, GABAergic and serotonergic neurotransmission ^(10)^. In patients with major depressive disorders, blood levels of miR-1202 were significantly lower compared to healthy controls ^(11)^. Furthermore, there was a relationship between blood miR-1202 expression and citalopram treatment response, with negative correlation between change in depression severity during the follow-up and change in miR-1202 expression ^(11)^.
  - **hsa-miR-146a/b-5p**: regulates genes involved in mitogen-activated protein kinase (MAPK) and Wnt signalling pathways ^(12)^. Blood expression of miR-146a/b in patients with major depressive correlates with response to treatment ^(12)^
  - **hsa-miR-22-3p**: might be associated with panic disorder ^(13)^ and in migraine without aura^(14)^, which pathophysiology involves dysregulation of the serotoninergic pathway^(15)^. miR-22-3p might participate in the expression of the expression of serotonin receptor-2c transcripts ^(13)^. In addition, extensive data also demonstrated the involvement of miR-22-3p in neuro-inflammation and brain response to ischemia ^(16)^. Most importantly, low miR-22-3p serum levels might be associated with poor response to antiseizure drugs in patients mesial temporal lobe epilepsy and clinical surrogate of the P2X7 purinoceptor expression in brain ^(17)^. Considering the potential role of the adenosine pathway in the pathology of epilepsy-related respiratory dysfunction^(18)^ and SUDEP^(19)^, this association between miR-22-3p and purinoceptors reinforces its interest in our panel.
  - **hsa-miR-21-5p**: involved in diverse pathophysiological processes relating to ischemia/reperfusion injury^(20)^. Blood levels of miR-21 have been proposed to be significant predictors of neurological outcome following cardiac arrest^(21)^, though another study did not confirm this observation^(22)^
  - **hsa-miR-106b-5p**: might play a role in epileptogenesis through regulating inflammation or apoptosis^(23)^. Blood levels of miR-106b were increased in patients with epilepsy in comparison with healthy controls^(23)^
  - **hsa-miR-199a-5p**: might target several pathways involved in epileptogenesis ^(24)^ and is upregulated in experimental temporal lobe epilepsy. Patients with temporal lobe epilepsy with hippocampal sclerosis have elevated levels in blood^(24)^.
  1. Identification of miRNAs of potential interest from a rat model of chronic epilepsy (Table 1)

This part of the study was conducted in Sprague Dawley rats using the same methods as previously reported^(1; 2)^ and was approved by the ethics committee of the University Claude Bernard Lyon 1 (approval 2018090611052453_v2). Briefly, pilocarpine-induced status epilepticus (Pilo-­SE) was triggered in 16 rats at the age of 7 weeks, resulting in occurrence 2-3 weeks after the SE of spontaneous recurrent seizures, as assessed by a protocol of visual tracking. 9-10 weeks post-SE, epileptic rats underwent plethysmography to assess ventilation. Ventilatory variables included respiratory frequency, estimated tidal volume and minute ventilation. Apnea events, defined as cessation of breath > 2 breath cycles, were also collected. According to the respiratory abnormalities previously reported in the same model^(1; 2)^, we separated epileptic rats (EpiRat) into two groups, those with respiratory dysfunction (ResD) and those without (noResD).

Following respiratory phenotyping (10-11 weeks post-SE), blood samples were collected in the lateral tail vein in 6 ReD and 6 noResD, as well as in 3 control rats. All samples were centrifuged (4°C, 1500 x *g*, 10 min) within 15 minutes of collection. Plasma was frozen in liquid nitrogen and stored at -80°C before being transferred to Laboratory for Epigenetics & Environment (CNRGH, Evry, France). The mean ± SD final volume of plasma samples was 483 ± 93.7 µL.

1. *Isolation of extracellular vesicles and extraction of total RNA*

500 µL of plasma samples (or the total available volume for the rat samples) were prefiltered through 0.8 µm Minisart™ NML Syringe Filters (Sartorius). Extracellular vesicles were isolated by membrane affinity using the ExoRNeasy midi kit (Qiagen) followed by total RNA extraction on a Qiacube liquid handler (Qiagen) using RNeasy MinElute spin columns (Qiagen) according to the manufacturer’s instructions and eluted into 15 µL ddH_2_0. 1.5 µL of the UniSp2, UniSp4 and cel-miR-39-3p spike-ins (RNA Spike-in Kit for RT, Qiagen) were included during the RNA purification for the human samples dedicated to qPCR analysis to control for the efficiency of RNA isolation.

1. *Rat model*

- **RNA library preparation and small RNA sequencing**

Small RNAs extracted from EVs were converted into barcoded cDNA libraries using the QIAseq miRNA Library Kit (Qiagen). The method integrates Unique Molecular Indices (UMIs) into the reverse transcription process, enabling unbiased miRNome-wide quantification of mature miRNAs by Next-Generation Sequencing (NGS) on the Illumina (San Diego, CA, USA) platform. The protocol was performed following the manufacturer’s instructions with slight modifications to be performed on a Freedom EVO150 liquid handling platform (Tecan, Männedorf, Switzerland).

Small RNAs, including mature miRNAs, were ligated to the QIAseq miRNA NGS 3’ Adapter and QIAseq miRNA NGS 5’ Adapter using QIAseq miRNA NGS Ligases (3’ and 5’ ligase). Subsequently, ligated miRNAs were hybridized to the miRNA NGS RT Initiator and reverse transcribed to cDNA using the QIAseq miRNA NGS RT Enzyme and QIAseq miRNA NGS RT Primer assigning a UMI to every miRNA molecule. After clean-up using the QIAseq miRNA NGS Beads with a 2.4X bead/cDNA ratio, cDNA libraries were indexed and amplified using HotStarTaq DNA Polymerase, QIAseq miRNA NGS ILM Library Forward Primer and index primers. The optimal number of PCR cycles (“N cycles”) was assessed by qPCR on a LightCycler 480 (Roche, Basel, Switzerland). The qPCR was performed with 10% volume of the purified cDNA, using the provided amplification reagents of the QIAseq miRNA Library Kit and 1X EvaGreen® Dye (Biotium, Hayward, CA, USA). The optimal cycle number corresponding to 1/3 of the highest fluorescent intensity was determined by plotting linear Rn. The subsequent PCR amplification of miRNA libraries was performed using the following conditions: denaturation for 15 minutes at 95°C, “N cycles” - 15 seconds at 95°C, 30 seconds at 60°C, 15 seconds at 72°C - and a final extension for 2 minutes at 72°C. Libraries were purified and a size selection was performed using the QIAseq miRNA NGS Beads, with 1X bead/cDNA ratio for the “1st Bead Selection” and 2,7X bead/cDNA ratio for the “2nd Bead Selection” to obtain library size distributions in the range of 150–250bp.

Library peak distribution was controlled and the average size was calculated using LabChip GX (PerkinElmer, Waltham, MA, USA). A miRNA-sized library is approximately 180 bp and a 188 bp peak corresponds to piRNA. Molarity of the miRNA library was determined by qPCR method using KAPA Library Quantification Kit (Roche) following the manufacturer’s recommendations. One sample of a healthy volunteer did not pass the quality control criteria for the small-RNA library preparation and was thus excluded from the subsequent analyses. Single-end miRNA sequencing was performed in a 12-plex on a HiSeq 4000 with sequencing read length of 75 bp (Illumina).

- **Bioinformatic analysis of small RNA sequencing data**

A modified version of the pipeline provided by Qiagen for use with the QiaSeq protocol was used to process raw data (fastq files to counts). Initially, raw reads were trimmed for adapter sequences using Cutadapt v.1.18. Reads were mapped using bowtie (v.1.1.2) against mature and stem-loop miRNA databases (miRbase v.22) as well as a proprietary in-house developed database, which takes natural variation of small RNA sequences into account. The aligned reads were counted by SAM tools v.0.1.19. The obtained read counts were passed for post-processing analysis, including counts normalization and outliers’ search.

The expected miRNA peak size of mapped reads of a size peaking at 22-23 nt was verified. Counts normalization to TPM (Transcripts Per Million) was performed during the post-processing. A threshold of TPM >10 was applied to filter out lowly expressed miRNAs and then assess if there are any significant differences in the overall distribution of particular samples using Principal Component Analyses (PCA) and normalized count statistics. The filtered count file was used for the differential analysis using Deseq2 (v.1.6.3), and in-house R scripts were used to interpret and evaluate the result.

1. **Analysis of expression profile of EV contained miRNAs by qPCR in Human samples**
   1. MicroRNA expression

First, a reverse transcription was performed for each sample using the miRCURY^®^ LNA^®^ RT kit (Qiagen) . 2 µL of the RNA eluate was used in addition to 2 µL 5x miRCURY RT reaction green buffer, 1µl of 10 x miRCURY RT enzyme, 0.5 µL UniSP6 spike-in and 4.5 µL ddH_2_O. The reaction mix was incubated for 60 min at 42°C, followed by 5 min at 95°C and hold at 4°C. The cDNAs were diluted thirty times in ddH_2_O before proceeding to the qPCR reaction. MiRNA Expression profile was determined by qPCR using the miRCURY^®^ LNA® SYBR® Green PCR kit (Qiagen). Following the manufacturer’s instructions, 3 µL diluted cDNA from each sample were used in combination with 5 µL 2x miRCURY SYBR Green master mix and 2 µL ddH_2_O. All cDNA mixes were distributed into custom 384-well Ready-to-Use PCR plates containing the assays for the selected miRNA and spike-in assays as well as the interplate calibrator UniSP3 (Qiagen) using a TECAN Freedom EVO^®^ 150 robot (Tecan, Männedorf, Switzerland). qPCR reactions were performed on a Lightcycler^®^ 480 V2 instrument (Roche Diagnostics, Meylan, France) using the following conditions: 95°C for 2 min, 40 amplification cycles at 95°C for 10s followed by 60s at 56°C.

- 1. Data analysis

The GenEx software (version 6, Exiqon) was used for the normalization of data and differential expression analysis. Data from each sample were combined, and inter-plate calibration and quality control was performed following the data analysis guide. All miRNAs with C_q_-values ≥ 35 of were removed from the analysis. As miRNAs selected as reference miRNAs from the rat model did not turn out to stable, we applied a global mean normalization, using the global mean of all Cq values < 35 for normalization, which has previously been shown to outperform other normalization strategy in terms of better reduction of technical variation and more accurate appreciation of biological changes ^(25; 26)^.

- 1. Statistics

Two analyses for the expression level of each miRNA included in the panel were conducted in parallel: (i) comparison between all patients with epilepsy and healthy subjects; and (ii) comparison between the two group of patients (PIH/noPIH).

In both analyses, miRNA expression levels were made using logistic regression and Mann-Whiney test. Adjustments for multiple comparisons were made using the Bonferroni method.

For each miRNA with differential expression levels between the two groups, receiver operating characteristics (ROC) curves were generated to assess its sensitivity and specificity and the area under the curves (AUCs) was calculated.

Data sharing statement: Raw data (CT values or processed log-2 fold ratios of miRNA expression data) are available from the corresponding authors upon request

**REFERENCES**

1. Kouchi H, Ogier M, Dieuset G, Morales A, Georges B, Rouanet JL, Martin B, Ryvlin P, Rheims S, and Bezin L. Respiratory dysfunction in two rodent models of chronic epilepsy and acute seizures and its link with the brainstem serotonin system. Scientific Reports 2022;12

2. Kouchi H, Smith J, Georges B, Cracknell F, Bezin L, and Rheims S. Serotonin 2C receptor in a rat model of temporal lobe epilepsy: From brainstem expression to pharmacological blockade in relation to ventilatory function. Epilepsia 2024;65:e125-30.

3. Richter DW, Manzke T, Wilken B, and Ponimaskin E. Serotonin receptors: guardians of stable breathing. Trends Mol Med 2003;9:542-48.

4. Petrucci AN, Joyal KG, Purnell BS, and Buchanan GF. Serotonin and sudden unexpected death in epilepsy. Exp Neurol 2020;325:113145.

5. Smith J, Richerson G, Kouchi H, Duprat F, Mantegazza M, Bezin L, and Rheims S. Are we there yet? A critical evaluation of sudden and unexpected death in epilepsy models. Epilepsia 2024;65:9-25.

6. Patodia S, Somani A, O’Hare M, Venkateswaran R, Liu J, Michalak Z, Ellis M, Scheffer IE, Diehl B, Sisodiya SM, and Thom M. The ventrolateral medulla and medullary raphe in sudden unexpected death in epilepsy. Brain 2018;141:1719-33.

7. Murugesan A, Rani MRS, Vilella L, Lacuey N, Hampson JP, Faingold CL, Friedman D, Devinsky O, Sainju RK, Schuele S, Diehl B, Nei M, Harper RM, Bateman LM, Richerson G, and Lhatoo SD. Postictal serotonin levels are associated with peri-ictal apnea. Neurology 2019;93:e1485-94.

8. Issler O, Haramati S, Paul ED, Maeno H, Navon I, Zwang R, Gil S, Mayberg HS, Dunlop BW, Menke A, Awatramani R, Binder EB, Deneris ES, Lowry CA, and Chen A. MicroRNA 135 is essential for chronic stress resiliency, antidepressant efficacy, and intact serotonergic activity. Neuron 2014;83:344-60.

9. Baudry A., Mouillet-Richard S., Schneider B., Launay J.-M., and Kellermann O. MiR-16 Targets the Serotonin Transporter: A New Facet for Adaptive Responses to Antidepressants. Science 2010;329:1537-41.

10. Pilc A, Chaki S, Nowak G, and Witkin JM. Mood disorders: regulation by metabotropic glutamate receptors. Biochem Pharmacol 2008;75:997-1006.

11. Lopez JP, Lim R, Cruceanu C, Crapper L, Fasano C, Labonte B, Maussion G, Yang JP, Yerko V, Vigneault E, El Mestikawy S, Mechawar N, Pavlidis P, and Turecki G. miR-1202 is a primate-specific and brain-enriched microRNA involved in major depression and antidepressant treatment. Nat Med 2014;20:764-68.

12. Lopez JP, Fiori LM, Cruceanu C, Lin R, Labonte B, Cates HM, Heller EA, Vialou V, Ku SM, Gerald C, Han MH, Foster J, Frey BN, Soares CN, Müller DJ, Farzan F, Leri F, MacQueen GM, Feilotter H, Tyryshkin K, Evans KR, Giacobbe P, Blier P, Lam RW, Milev R, Parikh SV, Rotzinger S, Strother SC, Lewis CM, Aitchison KJ, Wittenberg GM, Mechawar N, Nestler EJ, Uher R, Kennedy SH, and Turecki G. MicroRNAs 146a/b-5 and 425-3p and 24-3p are markers of antidepressant response and regulate MAPK/Wnt-system genes. Nat Commun 2017;8:15497.

13. Muiños-Gimeno Margarita, Espinosa-Parrilla Yolanda, Guidi Monica, Kagerbauer Birgit, Sipilä Tessa, Maron Eduard, Pettai Kristi, Kananen Laura, Navinés Ricard, Martín-Santos Rocío, Gratacòs Mònica, Metspalu Andres, Hovatta Iiris, and Estivill Xavier. Human microRNAs miR-22, miR-138-2, miR-148a, and miR-488 Are Associated with Panic Disorder and Regulate Several Anxiety Candidate Genes and Related Pathways. Biological Psychiatry 2011;69:526-33.

14. Tafuri E, Santovito D, de Nardis V, Marcantonio P, Paganelli C, Affaitati G, Bucci M, Mezzetti A, Giamberardino MA, and Cipollone F. MicroRNA profiling in migraine without aura: pilot study. Ann Med 2015;47:468-73.

15. Goadsby PJ, Holland PR, Martins-Oliveira M, Hoffmann J, Schankin C, and Akerman S. Pathophysiology of Migraine: A Disorder of Sensory Processing. Physiol Rev 2017;97:553-622.

16. Rastegar-Moghaddam SH, Ebrahimzadeh-Bideskan A, Shahba S, Malvandi AM, and Mohammadipour A. MicroRNA-22: a Novel and Potent Biological Therapeutics in Neurological Disorders. Mol Neurobiol 2022;59:2694-701.

17. Guerra Leal B, Barros-Barbosa A, Ferreirinha F, Chaves J, Rangel R, Santos A, Carvalho C, Martins-Ferreira R, Samões R, Freitas J, Lopes J, Ramalheira J, Lobo MG, Martins da Silva A, Costa PP, and Correia-de-Sá P. Mesial Temporal Lobe Epilepsy (MTLE) Drug-Refractoriness Is Associated With P2X7 Receptors Overexpression in the Human Hippocampus and Temporal Neocortex and May Be Predicted by Low Circulating Levels of miR-22. Front Cell Neurosci 2022;16:910662.

18. Bourgeois-Vionnet J, Jung J, Bouet R, Leclercq M, Catenoix H, Bezin L, Ryvlin P, and Rheims S. Relation between coffee consumption and risk of seizure-related respiratory dysfunction in patients with drug-resistant focal epilepsy. Epilepsia 2021;62:765-77.

19. Faingold CL and Feng HJ. A unified hypothesis of SUDEP: Seizure-induced respiratory depression induced by adenosine may lead to SUDEP but can be prevented by autoresuscitation and other restorative respiratory response mechanisms mediated by the action of serotonin on the periaqueductal gray. Epilepsia 2023;64:779-96.

20. Devaux Y, Stammet P, Friberg H, Hassager C, Kuiper MA, Wise MP, Nielsen N, and Biomarker subcommittee of TTM trial (Target Temperature Management After Cardiac Arrest NCT. MicroRNAs: new biomarkers and therapeutic targets after cardiac arrest. Crit Care 2015;19:54.

21. Stammet P, Goretti E, Vausort M, Zhang L, Wagner DR, and Devaux Y. Circulating microRNAs after cardiac arrest. Crit Care Med 2012;40:3209-14.

22. Gilje P, Gidlöf O, Rundgren M, Cronberg T, Al-Mashat M, Olde B, Friberg H, and Erlinge D. The brain-enriched microRNA miR-124 in plasma predicts neurological outcome after cardiac arrest. Crit Care 2014;18:R40.

23. Wang J, Yu JT, Tan L, Tian Y, Ma J, Tan CC, Wang HF, Liu Y, Tan MS, Jiang T, and Tan L. Genome-wide circulating microRNA expression profiling indicates biomarkers for epilepsy. Sci Rep 2015;5:9522.

24. Brennan GP, Bauer S, Engel T, Jimenez-Mateos EM, Del Gallo F, Hill TDM, Connolly NMC, Costard LS, Neubert V, Salvetti B, Sanz-Rodriguez A, Heiland M, Mamad O, Brindley E, Norwood B, Batool A, Raoof R, El-Naggar H, Reschke CR, Delanty N, Prehn JHM, Fabene P, Mooney C, Rosenow F, and Henshall DC. Genome-wide microRNA profiling of plasma from three different animal models identifies biomarkers of temporal lobe epilepsy. Neurobiol Dis 2020;144:105048.

25. Mestdagh P, Van Vlierberghe P, De Weer A, Muth D, Westermann F, Speleman F, and Vandesompele J. A novel and universal method for microRNA RT-qPCR data normalization. Genome Biol 2009;10:R64.

26. Chang KH, Mestdagh P, Vandesompele J, Kerin MJ, and Miller N. MicroRNA expression profiling to identify and validate reference genes for relative quantification in colorectal cancer. BMC Cancer 2010;10:173.
